# Assessing Health Impacts within Environmental Impact Assessments: An Opportunity for Public Health Globally Which Must Not Remain Missed

**DOI:** 10.3390/ijerph120101044

**Published:** 2015-01-20

**Authors:** Patrick Harris, Francesca Viliani, Jeff Spickett

**Affiliations:** 1 Menzies Centre for Health Policy, School of Public Health, Sydney Medical School, The University of Sydney, Room 322, Victor Coppleson Bld D02, Sydney, NSW 2006, Australia; 2 Centre for Primary Health Care and Equity, UNSW, Sydney, NSW 2052, Australia; 3 Public Health Consulting Services and Community Health Programs, International SOS, Radhuspladsen 16, DK 1550, Copenhagen, Denmark; E-Mail: Francesca.viliani@internationalsos.com; 4 School of Public Health, Curtin University, Kent St, Bentley Perth, Western Australia 6102, Australia; E-Mail: J.Spickett@curtin.edu.au; 5 WHO Centre for Environmental Health Impact Assessment. GPO Box U1987, Perth, Western Australia 6845, Australia

**Keywords:** health, Environmental Impact Assessment, sustainable development

## Abstract

Within the member states of the United Nations 190 of 193 have regulated Environmental Impact Assessments (EIA) which is a systematic process to prevent and mitigate the potential environmental impacts of industry development projects before these occur. However, the routine and comprehensive assessment of health impacts within EIAs remains underdeveloped. Focusing, as an example, on the risks to global health from the global shift in the mining industry towards Low and Middle Income Countries LMIC), this viewpoint details why connecting with EIA is an essential task for the health system. Although existing knowledge is out of date in relation to global practice we identify how health has been included, to some extent, in High Income Country EIAs and the institutional requirements for doing so. Using arguments identified by industry themselves about requiring a ‘social license to operate’, we conclude that EIA regulations provide the best current mechanism to ensure health protection is a core aspect in the decision making process to approve projects.

Environmental Impact Assessment (EIA) is the regulatory process within 190 of 193 member states of the United Nations to prevent and mitigate the potential environmental impacts of industry development projects before these occur [1]. EIA is recognized as a ‘globally significant decision tool’ [2] providing information and analysis that allows the public, proponents such as private or national industries, and regulators to understand, and have a dialogue about, the potential impacts of a project. Many EIA systems are built on the original 1969 National Environmental Protection Act (NEPA) in the United States, which explicitly included the words ‘human health’; Nigeria [3], for example, is a Lower Middle Income Country (LMIC) whose EIA system contains similar wording. Despite this, influencing the actual functioning of these systems, including under NEPA [4], to comprehensively incorporate health has, however, proven challenging and remains a missed opportunity for public health [5]. Focusing on the mining industry provides insight into why the global public health community must connect with EIA as a regulatory mechanism for protecting and improving health. 

The mining industry openly acknowledges a global shift in activity away from developed economies to ‘frontier markets’ and ‘rapidly emerging economies’, or LMICs. The International Council on Mining and Minerals, for example, has pointed out that ‘(Many of) the most important mining countries in terms of mineral production today are now emerging economies, often found south of the equator. Likewise the largest mines are now to be found in developing countries.’ [6]. Ernst and Young recently produced two reports about global mining industry trends, both explaining that ‘Rapid growth’ and ‘Emerging’ [7] economies are ‘future growth engines for the mining industry’ [8].

As these reports show, mining can play a role in shifting the economic growth of a country or region. Assessing these potential benefits—for example through increased local employment and economic growth—should remain a core focus of standard EIA practice as well as being connected to associated improvements in health. Additional positive community health impacts may be improved access to quality food, health care facilities and other infrastructure. However mining projects also pose enormous risks to health. Mining and minerals extraction has been shown in many different jurisdictions to impact on worker and community health: risks to workers include respiratory illness, injuries, cancers, and mental health; community health risk occur through exposure to air water, soil and noise pollution as well as disasters, and indirectly through migration including the transmission of communicable diseases such as HIV AIDS [9,10] and the Ebola virus [11]. Focusing in on specific industry activities, coal mining has been significantly associated with increased mortality and morbidity, hospitalizations, poor quality of life, and inequitable health problems for children and infants [12]. A recent global census of mining-related hazardous waste sites (n = 406), all in LMICs, with 7.5 million people estimated to be exposed] revealed that arsenic, lead, and mercury, all strongly associated with adverse health effects, comprised over 75% of the environmental risks at these sites [13].

The past decade has seen the private sector take the global lead on progressing health in EIA. The ‘Equator Principles’ are financial industry benchmarks for major project lending and identify “protection of community health, safety and security” to be potentially addressed in the EIA process [14]. These make direct reference for methodological guidance to the World Bank Group Standards and Guidelines on community health and safety [15]. Furthermore industry associations have developed sector specific guidance on Health Impact Assessment, most noticeably the International Council on Mining and Minerals [16] and OGP/IPIECA [17]. However these requirements have yet to be filtered down into existing regulatory systems within countries [18]. Further, the capacity of LMICs to develop effective EIA regulatory systems is likely to be limited even without the additional requirements for fully including human health [19]. 

One impediment to progress in gaining a better understanding of the issues is the current limited published knowledge base about including health in EIA in LMICs. A search of the research literature databases for the terms “Health” AND “Environmental Impact Assessments” AND (variations on) “LMICs” suggests only two (descriptive rather than empirical) articles focusing on health in EIA in LMICs; including health impact assessment in the EIA regulatory system in Nigeria [3], and including health in the EIA process in Thailand [20]. Reasons for this are varied. Currently the field is oriented towards developing practice with resources and capacity for systematic research yet to be viable. As with many practice based disciplines, for many EIA and health in EIA practitioners publication is not a top priority. The publications about health inclusion in EIAs are not accepted by many of the more prominent public health journals. Further, private sector confidentiality requirements mean EIAs including health are not readily available for systematic research. More broadly this limited knowledge corresponds with a global focus in health research on generating evidence about specific health conditions rather than regulatory systems and processes. Linking both types of knowledge has come to be known as implementation science and is gaining rapid interest from policy makers and researchers in recognition of the need for ‘sustained improvements to population health’ (p. 235; [21]); focusing on including health within EIA within broader approvals systems of environmental legislation is fertile ground for future implementation research. 

A publicly available knowledge base does exist from High Income Countries. This literature demonstrates the features of established EIA systems that are supportive and not supportive of assessing health impacts. However this knowledge concerns EIA systems which may not be easily transferable to LMIC contexts. The research is also, given developments over the past decade, increasingly out of date. 

This literature from High Income Countries does however reveal a number of core dimensions to including health in EIA. The word ‘health’ alone does not capture its full application in EIA [22]. Indeed the word ‘health’ is rarely included [4,22]. Changes to the bio-physical environment like air quality, noise and soil contamination, are considered (and socio-economic impacts are included less than bio-physical impacts) [4,22]. However EIAs rarely incorporate the assessment of pathways between these environmental exposures and health outcomes, use baseline health data to estimate community health impacts, or assess impacts on different population groups [4,5,22]. These findings are consistent with a worldwide review about health inclusion in different types of Impact Assessments, including EIA & Strategic Environmental Assessment [23]. There is now consensus that regulators, proponents and practitioners should initially consider the full range of health issues potentially affected by a project and then focus the actual impact assessment on those likely to have the most adverse or beneficial impacts [18]. 

Building on the global understanding that human health is central to sustainable development [24], the institutional goal for including health in EIA must be to equally prioritise community health alongside environmental protection, social development and economic growth in decisions about development projects. The literature (e.g., [3,5,18]) suggests what is required at various levels of regulatory systems to achieve this (see also [25]). At the top is political will and leadership within the environment and health sector, supported across government. Legislation is also required that explicitly includes health as a guiding principle or objective supported by regulatory frameworks and guidance. Implementation requires people on the ground with skills, experience as well as resources and support from within their organisations. Skills include technical skills such as risk assessment but also the ability to navigate and exchange ideas across disciplines. Without resources or organizational support it is impossible to make even legislative requirements a reality. Finally, community and other stakeholders require proactive engagement to link in with EIA public consultation processes.

EIA regulation exists in the majority of countries with a wide scope for including health issues comprehensively in the design, construction and operation of what is likely to be an explosion of resource development projects in LMICs. EIA has detractors and is not a silver bullet for public health; it can be cumbersome and be perceived as an administrative burden, the assumptions on which predictions are made can be opaque, it places power in the hands of public agencies rather than affected communities, and can lack regulatory teeth to enforce mitigation and changes to the design and operation of projects. These procedural problems do require EIA to be improved as a process. However it is likely that improving considerations of health issues can help to address these potential limitations. Just as the reports [7,8] by Ernst and Young point out, industry requires a social license to operate, with regulation being a space where community concerns can be realized. Communities should be increasingly concerned and active about the health consequences of increased industry activity, and EIA systems are the regulatory point where these concerns must be taken on board by industry. Giving greater consideration to the health impacts of a development project within the current EIA processes will provide the best opportunity to consider health impacts within the current approvals processes and regulatory systems. The ultimate challenge however is whether the health systems in those countries can recognize, work with and leverage support within EIA as a force for protecting and improving public health.
